# Percutaneous vascular plug in management of an acquired broncho pleural cutaneous fistula

**DOI:** 10.1186/s42155-024-00508-9

**Published:** 2025-03-17

**Authors:** Ajay Alex, Praveen A, Niwin George, Vinu C V, Radhika Devi B, Neetha Jose

**Affiliations:** 1 Department of Imaging and Interventional Radiology, Sree Chitra Tirunal Institute for Medical Sciences and Technology, Trivandrum, Kerala India; 2 Department of Radiodiagnosis and Interventional Radiology, Government Medical College, Trivandrum, Kerala India; 3 Department of Cardiothoracic and Vascular Surgery, Government Medical College, Trivandrum, Kerala India; 4 Department of Anaesthesiology, Government Medical College, Trivandrum, Kerala India

**Keywords:** Broncho pleural cutaneous fistula, CERA vascular plug

## Abstract

**Background:**

Bronchopleural fistula (BPF) / broncho pleural cutaneous fistula is an abnormal communication between the peripheral bronchial tree and pleural space which can further also open to the skin surface. It is associated with significant morbidity and mortality in addition to poor quality of life. Management requires a multidisciplinary approach with careful evaluation to choose the best approach to treatment.

**Case presentation:**

A 36-year-old male presented with a left chest wall tumor with multiple surgeries and CT revealing a left apico-posterior segment broncho pleural cutaneous fistula. Various options for the management of the BPCF including surgery and bronchoscopic occlusion were considered however an IR approach was planned. Plan was for vascular plug occlusion with/without glue embolization of the apico-posterior segmental bronchus. A 6F sheath was placed under direct vision and a 12 mm CERA plug was deployed. After plain plug occlusion, there were no signs of air leak. Various options for management including surgery and bronchoscopy procedures are limited in patients with poor pulmonary reserve. The IR approach offers the advantage of doing the procedure under real-time fluoroscopy, and no airway compromise. However, literature describes the use of glue to seal the interstices of the device which if not sealed was a cause of recurrence later. In our case, we report the percutaneous use of a CERA vascular plug as the sole device, especially since it has a polytetrafluoroethylene (PTFE) membrane which ensures occlusion, in addition to its titanium nitride coating which improves epithelialization. This ensures sustained occlusion as the sole agent, unlike other devices including the Amplatzer vascular plug.

**Conclusions:**

This highlights the relatively easy percutaneous route and the first report of CERA vascular plug usage for managing a case of broncho pleural cutaneous fistula.

**Graphical abstract:**

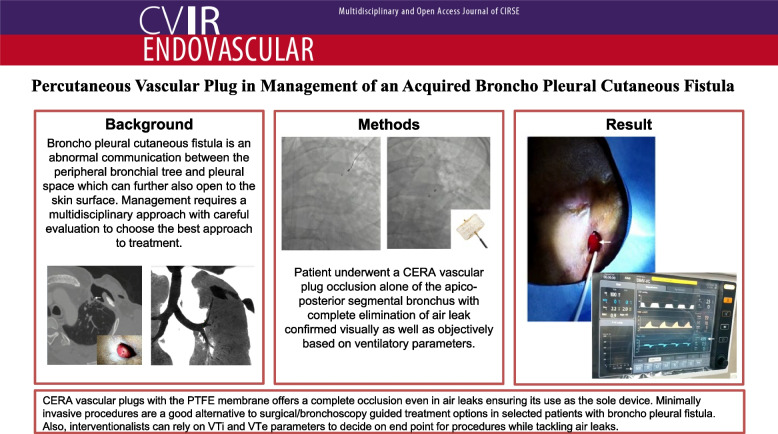

**Supplementary Information:**

The online version contains supplementary material available at 10.1186/s42155-024-00508-9.

## Background

Bronchopleural fistula (BPF) / broncho pleural cutaneous fistula is an abnormal communication between the peripheral bronchial tree and pleural space which can further also open to the skin surface. Some authors consider it a double fistula [1]. They occur usually following an infection, surgery, or tumor in relation to the peripheral lung, pleura, or overlying chest wall structures. It is associated with significant morbidity and mortality in addition to poor quality of life. Management requires a multidisciplinary approach with careful evaluation to choose the best approach to treatment.

## Case presentation

A 36-year-old male presented with a left chest wall tumor that had pleural infiltration in 1998, for which patient underwent excision. The histopathological report was of a leiomyosarcoma. Further, the patient on follow-up in 2018 developed a recurrence for which he underwent a repeat surgery. However, 1 month following surgery he developed a wound site infection with pus drainage. He was on surgical follow-up with further, the skin site also giving away. He was attempted for medical management on antibiotics and occasional local site debridement for nearly 6 months with no success.

**Fig. 1:**
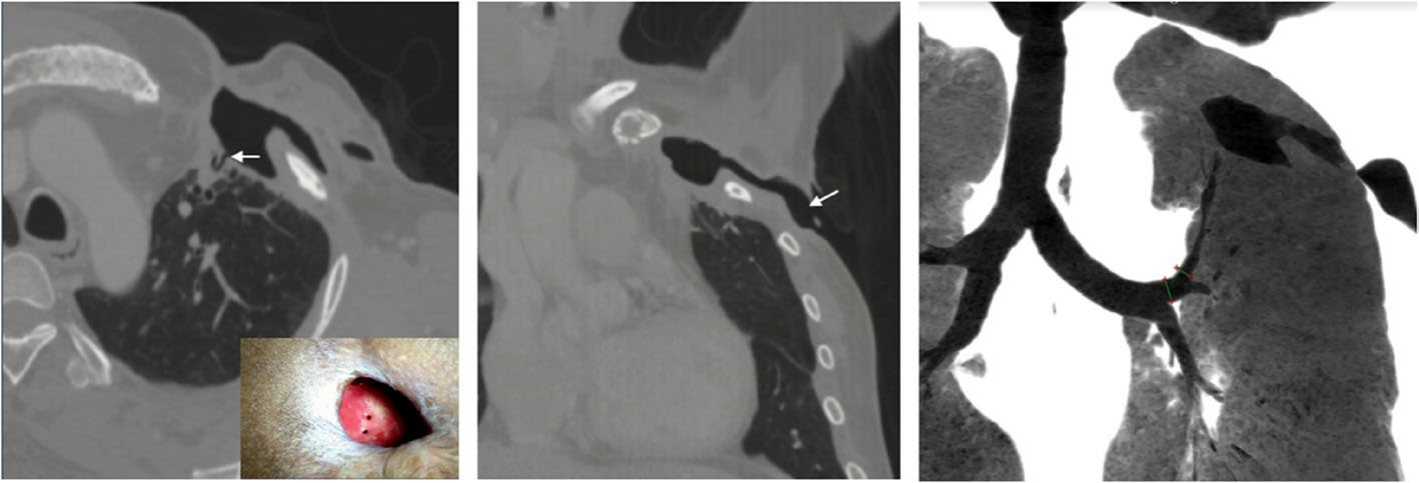
**a** Axial computed tomography (CT) lung window showing the peripheral bronchopleural fistula (arrow). Inset depicts the skin site over the patient's left chest with two openings. **b** Coronal CT lung window showing the pleural to cutaneous component(arrow) of the fistula. **c** Coronal CT Minimum intensity projection image showing the apicoposterior segment bronchus extending to the pleural surface and further to the skin

Computed tomography (CT) revealed left upper lobe peripheral bronchioles involving the apico-posterior segment opening into the pleural cavity via two openings and further to the cutaneous level (Fig. 1). On clinical examination, the cutaneous opening with the peripheral BPF was also seen directly (Fig. 1a).

A multidisciplinary team consisting of cardiothoracic surgeons, anaesthetists, and interventional radiologists, considered various options for the management of the BPF. Open surgery was not preferred given the patient's poor general condition and history of previous multiple surgeries. Bronchoscopy-guided occlusion was also deferred given the patient's poor pulmonary reserve especially since the bronchoscope will take up space within the bronchus and compromise oxygenation further. Finally, a decision was made for the interventional radiological approach. Plan was for vascular plug occlusion with glue embolization of the proximal part of the apicoposterior segmental bronchus.

The procedure was done under general anesthesia. The skin site was scrubbed with a 5% povidone-iodine microbicidal solution and sterile drapes were placed. Further, 10 ml sterile saline was instilled into the skin defect to demonstrate an air leak due to the BPF.

**Fig. 2:**
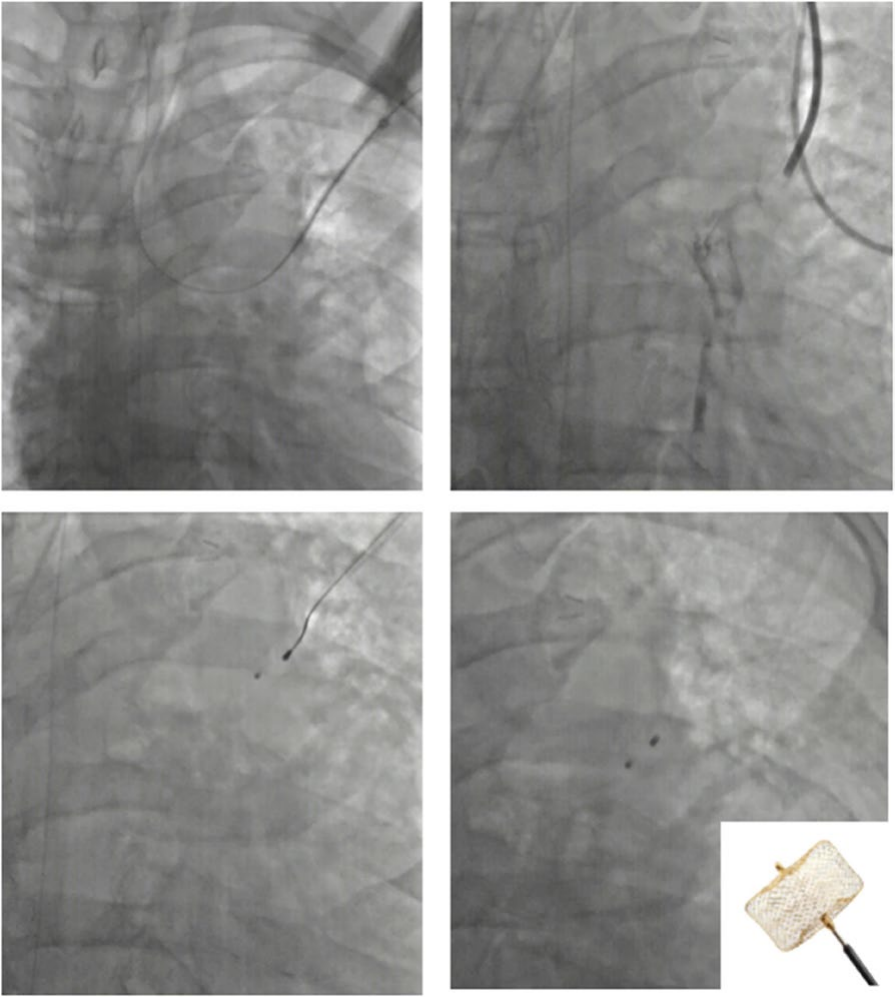
Fluoroscopy images of the procedure **a** 0.018″ wire being passed into the left main bronchus via the percutaneous route under direct visual approach, and 6F sheath being placed over the wire. **b** Nonionic iodinated contrast instilled to delineate the bronchial anatomy. **c** Vascular plug being deployed through the sheath after confirming the site of occlusion. **d** Final image after the plug is detached from the wire. *Inset* CERA vascular plug with PTFE membrane

**Fig. 3:**
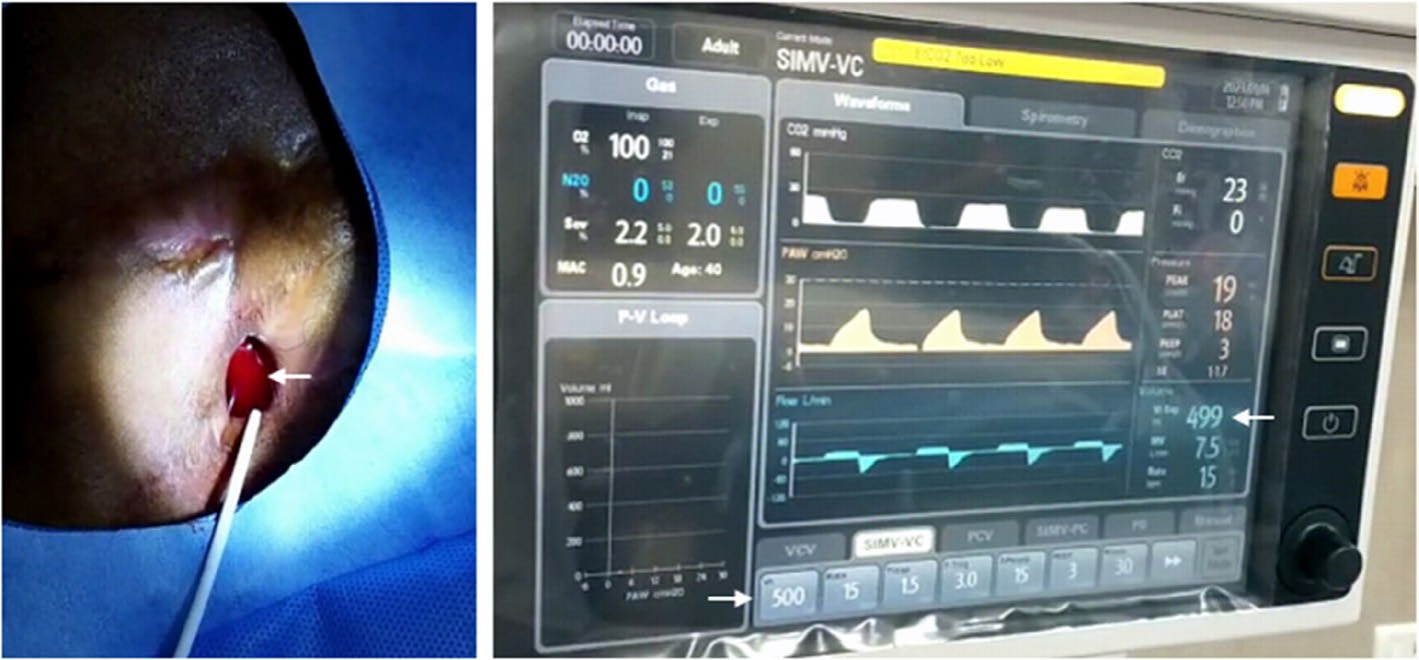
**a** No air leak as evidenced by absence of bubbling after plug deployment with saline (arrow) filled over the orifice. Sheath is seen withdrawn with only the vascular plug wire within. **b** Ventilator parameters showing corresponding tidal volume parameters (arrows) confirming objectively the absence of leak

Further 0.018″ wire was passed under direct vision into the peripherally seen bronchiole. Under fluoroscopy, the same was negotiated into the left main bronchus and further into the carina (Fig. 2a). Further, a 6F short sheath was introduced over the wire into the segmental bronchus slowly. To delineate the bronchial anatomy 2–3 ml of non-ionic iodinated contrast (Iohexol) was instilled (Fig. 2b). Further, after delineating the required proximal site of occlusion, a 12 mm CERA™ vascular plug (Lifetech Scientific, Shenzhen) was deployed. 3D cone beam CT was also taken to confirm adequate positioning. Further, a repeat instillation of 10 ml sterile 0.9% normal saline was done to check for air leaks. No bubbling was detected (Fig. 3a). Additionally, ventilatory parameters revealed corresponding tidal volume of 500(VTi)/499(VTe) which was before the procedure at 500 (VTi) /392 (VTe) (Fig. 3b).

After plug occlusion, the patient was extubated and followed up clinically with the patient being discharged after 5 days with no signs of air leak. The patient had no migration of the plug on follow-up and no leak. Patient gradually showed epithelialisation over the defect on the lung surface with no leak at 6 month follow up.

## Discussion

Broncho pleural cutaneous fistula is a rare complication that mostly develops postoperatively [2]. Initial management involves oxygen supplementation and chest tube insertion in addition to control of infection. Persistent fistulas with significant air leaks require management surgically with pneumonectomy. Lately, there have been various alternative ways of management mainly via a bronchoscopic route with the use of plug devices, the largest series described by Katoch et al. [3]. Bronchoscopic intervention may not be tolerated by many patients as the bronchoscope itself will occupy a large portion of the airway lumen during the procedure, compromising intraprocedural oxygen maintenance. The interventional radiological approach to managing BPF is also described which offers the advantage of doing the procedure under real-time fluoroscopy, and approach via either bronchial route or percutaneous route. The percutaneous approach of doing CT or ultrasound-guided glue or autologous blood patch has also been reported [4, 5]. The intrabronchial route after intubation or through a small puncture at the tracheostomy site through the trachea has also been described for closure using a duct occluder device and coils [6, 7]. However, most of the described literature when using devices via fluoroscopy guidance describes the use of glue to seal the interstices of the device which if not sealed was a cause of recurrence later. In our case, we report the percutaneous use of a CERA vascular plug as the sole device, especially since it has a polytetrafluoroethylene (PTFE) membrane which ensures occlusion, in addition to its titanium nitride coating which improves epithelialization. This ensures sustained occlusion as the sole agent, unlike other devices including the Amplatzer vascular plug. This case highlights the relatively easy percutaneous route and the first report of CERA vascular plug usage for managing a case of broncho pleural cutaneous fistula.

## Supplementary Information

Supplementary Material 1.

Supplementary Material 2.

Supplementary Material 3.

## Data Availability

All data generated or analysed during this study are included in this published article and its supplementary information files.

## Abbreviations

BPF: Bronchopleural fistula
CT: Computed tomography
PTFE: Polytetraflouroethylene
VTi: Tidal Volume Inspiratory
VTe: Tidal Volume Expiratory
